# Metaboloepigenetics in cancer, immunity, and cardiovascular disease

**DOI:** 10.1093/cvr/cvac058

**Published:** 2022-04-07

**Authors:** Samuel T Keating, Assam El-Osta

**Affiliations:** Department of Biology, University of Copenhagen, Copenhagen DK-2200, Denmark; Department of Diabetes, Central Clinical School, Monash University, Melbourne, Victoria 3004, Australia; Epigenetics in Human Health and Disease Laboratory, Central Clinical School, Monash University, Melbourne, Victoria 3004, Australia; Department of Medicine and Therapeutics, The Chinese University of Hong Kong, Sha Tin, Hong Kong SAR; Hong Kong Institute of Diabetes and Obesity, Prince of Wales Hospital, The Chinese University of Hong Kong, 3/F Lui Che Woo Clinical Sciences Building, 30-32 Ngan Shing Street, Sha Tin, Hong Kong SAR; Li Ka Shing Institute of Health Sciences, The Chinese University of Hong Kong, Hong Kong, Sha Tin, SAR; Faculty of Health, Department of Technology, Biomedical Laboratory Science, University College Copenhagen, Copenhagen, Denmark

**Keywords:** Metabolism, Epigenetics, Metaboloepigenetics, Glycolysis, Trained immunity, Cardiovascular disease, Diabetes

## Abstract

The influence of cellular metabolism on epigenetic pathways is well documented but misunderstood. Scientists have long known of the metabolic impact on epigenetic determinants. More often than not, that title role for DNA methylation was portrayed by the metabolite *S*-adenosylmethionine. Technically speaking, there are many other metabolites that drive epigenetic processes that instruct seemingly distant—yet highly connect pathways—and none more so than our understanding of the cancer epigenome. Recent studies have shown that available energy links the extracellular environment to influence cellular responses. This focused review examines the recent interest in epigenomics and casts cancer, metabolism, and immunity in unfamiliar roles—cooperating. There are not only language lessons from cancer research, we have come round to appreciate that reaching into areas previously thought of as too distinct are also object lessons in understanding health and disease. The Warburg effect is one such signature of how glycolysis influences metabolic shift during oncogenesis. That shift in metabolism—now recognized as central to proliferation in cancer biology—influences core enzymes that not only control gene expression but are also central to replication, condensation, and the repair of nucleic acid. These nuclear processes rely on metabolism, and with glucose at centre stage, the role of respiration and oxidative metabolism is now synonymous with the mitochondria as the powerhouses of *metaboloepigenetics*. The emerging evidence for metaboloepigenetics in trained innate immunity has revealed recognizable signalling pathways with antecedent extracellular stimulation. With due consideration to immunometabolism, we discuss the striking signalling similarities influencing these core pathways. The immunometabolic-epigenetic axis in cardiovascular disease has deeply etched connections with inflammation, and we examine the chromatin template as a carrier of epigenetic indices that determine the expression of genes influencing atherosclerosis and vascular complications of diabetes.

## 1. Introduction

More than 20 years have passed since the landmark publication of the human genome sequence.^1^ While researchers have benefited enormously from an improved understanding of the genetic sequence, many of the promises of the genetic revolution remain unfulfilled, particularly with regard to the complexity of phenotypic traits and the propensity to develop certain diseases.^2,3^ As researchers learned that humans have fewer genes than a banana,^4^ it was clear that genome size is unrelated to organism complexity. Instead, we have begun to appreciate that phenotypic traits can associate not only with nucleotide sequence but also with chemical modifications that occur on the DNA template and the proteins with which it interacts. Closely following this realization came a broader awareness of how cells with identical genetic content could exhibit different regulation of gene activity.

Modern interpretations of epigenetics have focused on the covalent chemical modifications of chromatin—the dynamic complex of DNA and proteins (mainly histones)—that support transcriptional regulation via structural adaptation.^5^ Collectively, these modifications are called epigenetic because they influence phenotypes without altering the genetic code and have been shown to be transmitted through cell division by various mechanisms.^6–11^ Three distinct yet functionally related categories of epigenetic mechanisms have been described: (i) chemical modification of DNA bases, (ii) post-translational modification (PTM) of the tails of histone proteins, and (iii) the regulation of gene expression by non-coding RNA. Despite significant progress in the field, knowledge of how the main epigenetic players are regulated remains incomplete.

A substantial number of findings connecting energy metabolism with epigenetic control of gene expression support the recent emergence of what some in the field are calling ‘metaboloepigenetics’.^12–14^ Many enzymes that write or erase modifications on the chromatin use metabolites as substrates or cofactors in their epigenetic reactions, connecting metabolic information with transcription.^15^ Changes in the concentrations of specific metabolites are therefore purported to provide signalling cues for the continual adjustment of gene expression by influencing chromatin dynamics. What is emerging is a complex interplay between intracellular metabolism and chromatin modifications, which is providing an extra dimension to our understanding of gene regulation in health and disease.

The current understanding of metaboloepigenetics has its origins in cancer, a disease characterized by the derangement of metabolic and epigenetic programmes.^16,17^ More recent findings have shed light on the changes in intracellular metabolic pathways that support the altered function of immune cells via epigenetic reprogramming.^18–20^ Furthermore, researchers have begun to consider the impact of metaboloepigenetics on atherosclerosis and cardiovascular disease (CVD).^21–23^

## 2. Metabolism drives epigenetic processes: lessons from cancer

Gene expression is primarily regulated by the accessibility of DNA to transcription factors (TFs) and transcriptional machinery. Key to this regulation is chromatin, a dynamic assembly of DNA and regularly spaced nucleosomes (comprised of histone proteins) that controls transcription by structural adaptation and genome compartmentalization.^15^ Compacted chromatin impedes binding of the transcriptional machinery to the DNA by occluding its access to regulatory elements such as promoters and enhancers. On the other hand, an open chromatin structure facilitates the loading of transcriptional machinery. Supporting the gene-regulating functions of TFs is a multitude of PTMs to the N-terminal tails of histones as well as modifications to DNA bases. Some of these modifications have direct effects on chromatin structure by altering charge states of histones and their affinity for DNA. Others provide docking sites for the recruitment of multi-protein chromatin remodelling complexes. For a comprehensive discussion of the enzymes that write, erase, and interpret chromatin modifications, we recommend a recent review article by Zhao *et al*.^5^

At the most basic level, a specific chromatin modification is dependent on the relative expression, stability, and competing activities of epigenetic writers and erasers. In recent years, cellular metabolism has emerged as an important determinant of many epigenetic reactions, primarily because numerous epigenetic enzymes require specific metabolites or products from metabolic pathways as cofactors to perform their chromatin modifying functions. Several different metabolic pathways are used by cells to generate adequate energy for survival and to produce biosynthetic intermediates that support cellular growth and proliferation. These distinct metabolic pathways are closely connected by the use of common fuel inputs and a reliance on products from one pathway to feed into alternative pathways as synthetic precursors.^24^ While a certain degree of overlap exists between metabolic pathways in terms of the chromatin-regulating metabolites that they produce, individual pathways produce metabolites that intersect epigenetic pathways in specific ways. This means that large metabolic shifts can have profound effects on epigenetic regulation and gene expression profiles.^25^

Metabolic reprogramming has been recognized as a hallmark of cancer transformation since Otto Warburg first described aerobic glycolysis in tumours almost a century ago.^26^ Human tumours harbour global epigenetic abnormalities^27^ and mutations in genes encoding epigenetic enzymes are increasingly recognized in cancer.^28^ Unsurprisingly, many of the known metaboloepigenetic connections were discovered in the metabolically deranged milieu of cancer,^29^ which spawned the concept of the oncometabolite—a metabolic intermediate whose abnormal accumulation triggers oncogenic signalling and tumourigenesis.^30^

### 2.1 Metabolites modulate the methylome

Methylation of cytosine bases is the most well-characterized epigenetic mark.^31^ The addition of a methyl group to the fifth carbon of a cytosine base (5-methylcytosine, 5mC) is catalysed by DNA methyltransferase enzymes (DNMTs) primarily at cytosines adjacent to a guanines (cytosine-phosphate-guanine, CpG). The vast majority of CpG dinucleotides in mammalian genomes are maintained in a methylated state with the exception of regions of high CpG density called CpG islands located close to gene promoters. A more recent discovery is the mechanism of cytosine demethylation by ten-eleven translocation (TET) dioxygenases that oxidize the methyl group of 5mC to yield 5-hydroxymethylcytosine (5 hmC) and other oxidized methylcytosines.^32–34^ DNA methylation is primarily associated with gene silencing by two main mechanisms: (i) the occlusion of DNA binding proteins such as TFs that act as transcriptional activators, and (ii) by providing a recognition site for methyl-binding proteins such as methyl-CpG binding protein 2, which recruit transcriptional corepressor complexes that reconfigure the chromatin landscape, rendering it inaccessible to the transcriptional machinery.^35^ DNA methylation has a dual role in cancer: hypermethylation inhibits tumour suppressor genes, whereas hypomethylation activates oncogene expression. In general, cancer cells display a global loss of CpG methylation juxtaposed against locus-specific hypermethylation at CpG islands.^36^ Paradoxically, under some circumstances, DNA hypermethylation has also been shown to enhance gene expression in cancer and recently reviewed.^37^

When assigned to the tails of histones, the methyl modification provides a greater degree of flexibility with regard to its impact on gene expression. Both lysine and arginine residues are sites of histone methylation: arginine residues can be mono-methylated or di-methylated and lysine residues can be mono-, di-, or tri-methylated. This is important because variably methylated histones are differentially distributed across chromatin to distinguish gene regulatory elements. For example, H3 histones tri-methylated at lysine 4 (H3K4me3) are enriched at promoters of transcribed genes, whereas H3 histones mono-methylated at lysine 4 (H3K4me1) are predominantly enriched at distal enhancers.^38,39^ However, the key determinant of the effect of histone methylation on chromatin structure is the position of the modified residue within the amino sequence of the histone tail. In addition to the transcriptionally permissive methylated state of H3K4, other key sites of histone lysine methylation include lysine 9 and lysine 27 on H3 histones, which are associated with gene repression. Methyl modifications were considered more stable than other histone marks until the discovery of lysine-specific histone demethylase 1 (LSD1), which enzymatically removes methyl groups from H3K4. This led to the identification of the jumonji C (JmjC) domain as a key catalytic component of a broad catalogue of histone lysine demethylase (KDM) enzymes.^40^ Thus, levels of histone lysine methylation are determined in part by the relative expression and activity of lysine methyltransferases (KMTs) and KDMs. Like DNA methylation, histone methylation can also provide sites for the recruitment of effector protein complexes that recognize distinct methyl modifications via specialized domains.^41^

Most methyltransferases transfer a methyl group from *S*-adenosylmethionine (SAM), which is synthesized from adenosine triphosphate (ATP) and methionine. The methylation reaction generates another metabolite called *S*-adenosylhomocysteine (SAH), which is a potent inhibitor of methyltransferases. Therefore, the intracellular SAM:SAH ratio, which is regulated by methionine, threonine, and serine metabolism, as well as dietary intake of methyl-donating nutrients such as folate and vitamin B, is considered to be an indicator of cellular methylation potential.^42,43^ In addition, cells require one-carbon units for nucleotide synthesis and redox reactions. Because these pathways support the enhanced proliferation of cancer cells, drugs that target one-carbon metabolism such as the anti-folate methotrexate have long been used in cancer therapy.^44^ Glycine N-methyltransferase (GNMT) deficiency is a rare condition leading to SAM accumulation.^45^*Gnmt* knockout mice exhibited a more than 40-fold increase in hepatic SAM,^45^ which led to DNA hypermethylation and transcriptional silencing of tumour suppressor genes and was associated with increased incidence of hepatocellular carcinoma.^46^ As a general hallmark of cancer, methionine addiction is targeted in cancer therapy by methionine restriction, which results in the depletion of SAM^47^ and cell cycle arrest.^48^ Recent studies have begun to investigate the therapeutic potential of methionine restriction in combination with inhibitors of SAM synthesis and DNA methylation.^49^

### 2.2 Mitochondria are the powerhouses of metaboloepigenetics

**Figure 1:**
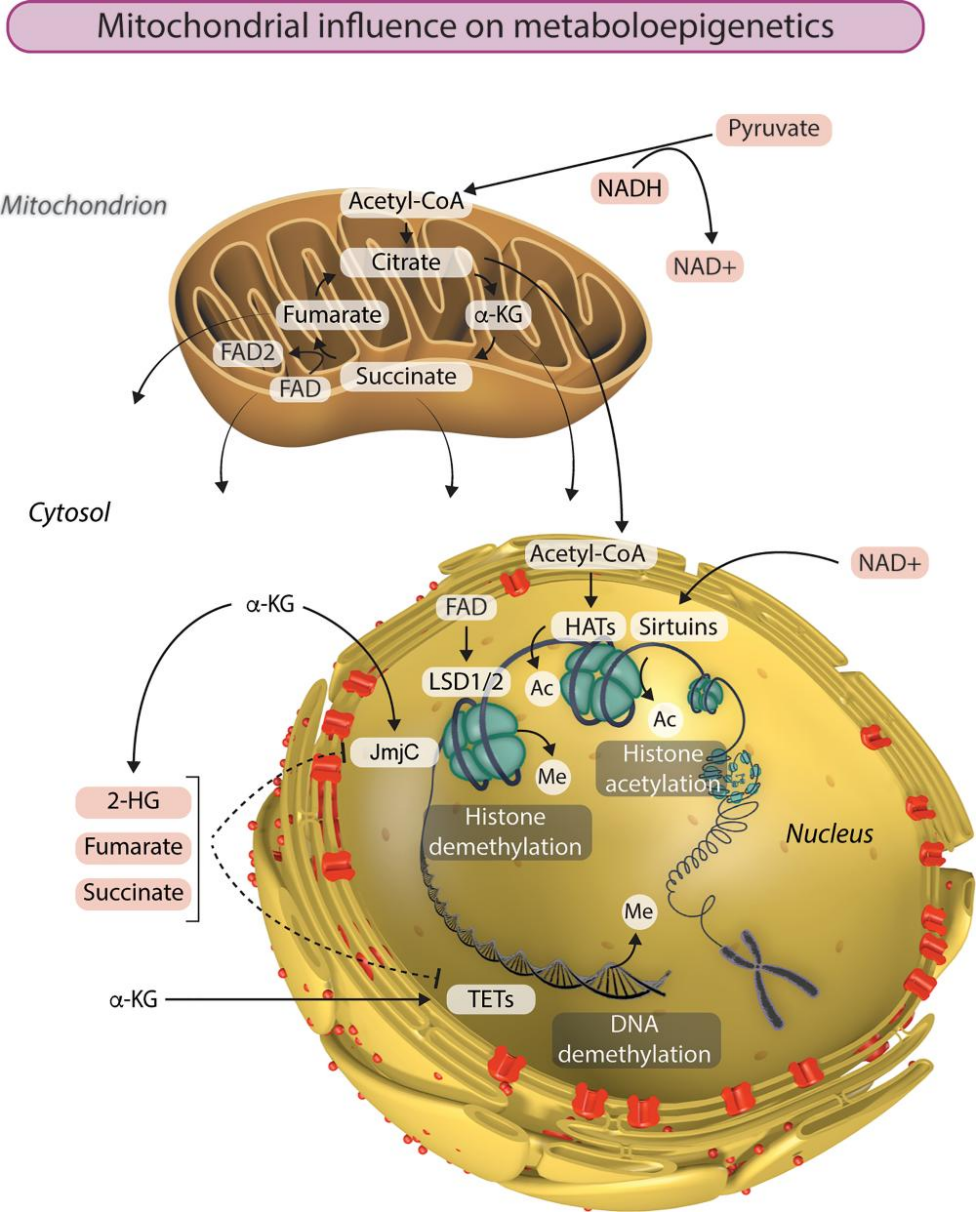
Mitochondria are the powerhouses of metaboloepigenetics. Mitochondrial function, and particularly the TCA cycle, provides the intermediate metabolites essential to the generation and modification of epigenetic marks in the nucleus. Histone acetylation by histone acetyltransferases (HATs) is dependent on the availability of acetyl-CoA. On the other hand, histone acetylation can be removed by a class of NAD^+^-dependent histone deacetylases called sirtuins. LSD1 and LSD2 catalyse demethylation of mono- and di-methylated lysine residues using FAD as a cofactor. The availability of α-ketoglutarate (α-KG) influences histone and DNA demethylation by JmjC and TET enzymes respectively. Succinate, fumarate, and 2-hydroxyglutarate (2-HG) can also influence the epigenetic landscape by inhibiting α-KG-dependent histone and DNA demethylation. Ac, acetyl group; Me, methyl group.

The removal of methyl groups from DNA and histones plays a critical role in shaping the epigenome. In recent years, the tricarboxylic acid (TCA) cycle has taken centre stage as a source of metabolites than influence chromatin demethylation, primarily through its effects on the activity of JmjC KDMs and TETs (*Figure 1*). Both of these classes of dioxygenases are dependent on α-ketoglutarate (α-KG) as a cofactor in reactions that remove methyl groups from histones and DNA, respectively. This key TCA cycle intermediate is produced from isocitrate by isocitrate dehydrogenase or by anaplerotic synthesis from glutamate. Isocitrate dehydrogenase (IDH) genes *IDH1* and *IDH2* are the most frequently mutated metabolic genes identified in human cancers.^50^ In addition to losing normal catalytic activity for the production of α-KG, mutant IDH1 and IDH2 gain the function of catalysing the reduction of α-KG to produce 2-hydroxyglutarate (2-HG), which has been described as an oncometabolite capable of stimulating proliferation and suppressing differentiation.^51–53^ Due to structural similarity, 2-HG can bind to and function as a competitive inhibitor of α-KG-dependent KDMs and TETs.^17^ Interestingly, *TET2* is also frequently mutated in acute myeloid leukaemia, in which *IDH1/2* mutations are common.^54^ Similarly, aberrant DNA methylation profiles and gene expression patterns were observed in AML with either *IDH1/2* or *TET2* mutations, indicating that TET2 is a pathologically relevant target of 2-HG. Furthermore, glioma samples harbouring mutant IDH1 accumulate significantly lower 5hmc and significantly higher 5 mc than those containing wild-type IDH1, in accordance with reduced TET activity.^17^

Also within the TCA cycle, succinate dehydrogenase (SDH, comprised of four subunits: SDHA, SDHB, SDHC and SDHD) catalyses the oxidation of succinate to fumarate and uses the electrons to reduce ubiquinone to ubiquinol in the electron transport chain. The next enzyme in the TCA cycle, fumarate hydratase (FH), catalyses the reversible hydration of fumarate to malate. Succinate and fumarate are competitive inhibitors of α-KG-dependent dioxygenases. Knockdown of FH and SDH results in elevated intracellular levels of fumarate and succinate, respectively, both of which broadly inhibit the activity of α-KG-dependent dioxygenases.^55^ On the other hand, ectopic expression of tumour-derived FH and SDH mutants inhibits histone demethylation and hydroxylation of 5 mC. Accordingly, SDH deficiency underlies pervasive DNA hypermethylation in both gastrointestinal stromal tumours and paraganglioma/pheochromocytoma tumours.^56^ This part of the TCA cycle can further influence histone methylation by modulating the activity of another class of KDMs—the amine oxidases LSD1 and LSD2 that catalyse demethylation of mono- and di-methylated lysine residues using flavin adenine dinucleotide (FAD) as an essential cofactor.^57^ The conversion of succinate to fumarate by SDH reduces FAD to FADH_2_, thereby altering the cellular availability of FAD, which can have consequences for histone methylation.^58^

Very recently, Liu *et al*.^59^ identified a nonclassical TCA (nTCA) cycle in the nuclei of mouse and human cells. All components of the classical TCA cycle except for SDH are present in the nucleus, where they catalyse TCA cycle-related biochemical reactions. By functionally linking the nTCA cycle to epigenetic regulation, chromatin dynamics and gene expression, the authors propose that a self-sustaining nTCA cycle is implemented to supply and consume metabolites involved in the dynamics of DNA and histone modifications.^59^

### 2.3 Warburg metabolism: glycolysis and glutaminolysis

The Warburg effect is a signature of metabolic change in cancer. This metabolic rewiring is defined by increased glucose uptake and the fermentation of glucose to lactate, even in the presence of functioning mitochondria and oxygen, with a concomitant decrease in oxidative phosphorylation (OxPhos).^26^ While aerobic glycolysis generates much less ATP (2 molecules of ATP per unit of glucose) than does OxPhos (36 molecules of ATP per unit of glucose), switching to Warburg metabolism allows cancer cells to (i) more rapidly meet the bioenergetic demands of proliferation (glycolysis can be ramped up by relatively few enzymes, whereas increased OxPhos most likely requires mitochondrial biogenesis), and (ii) convert nutrients more efficiently into the biomass (nucleotides, amino acids, and lipids) needed to produce a new cell.^60^ These changes are largely regulated by HIF1α, a key TF for the expression of genes involved in glycolysis and the induction of pseudohypoxia in cancer cells,^61^ as well as mTOR signalling, which senses nutrients to support cell growth.^62^

The metabolic shift inexorably leads to the accumulation of methylgyoxyl (MGO), primarily through the fragmentation of triose phosphate intermediates of glycolysis.^63^ This toxic and highly reactive dicarbonyl spontaneously glycates lipids and proteins and is a potent inducer of advanced glycation end-products. Accumulation of MGO is limited by the glutathione-dependent enzyme, Glyoxalase 1, which is overexpressed in many cancers as a defence strategy against MGO cytotoxicity.^64,65^ On the other hand, recent evidence ^66,67^ indicates a hormetic effect, in which low doses of MGO support an adaptive response in cancer cells while high doses cause apoptosis.^68^ In addition to many cellular proteins, MGO-derived adducts were recently found to occur abundantly on histone tails, particularly arginine residues, under conditions of metabolic stress.^69^ These non-enzymatic modifications alter chromatin architecture by competing with other histone modifications, disrupt nucleosome assembly and chromatin fibre compaction, and down-regulate transcription.^70^ Protein arginine deaminase 4 (PAD4) was found to antagonize histone MGO-glycation by removing glycation adducts from arginine residues, as well as by converting unmodified arginine to citrulline, thereby protecting them from glycation. Indeed, PAD4 is overexpressed and citrulline is upregulated in breast tumours.^71^ These findings suggest an additional mechanistic link between metabolism and cancer epigenetics that requires more research attention.

Under normal metabolic conditions, pyruvate is fed into the TCA cycle, which generates ATP by OxPhos while at the same time providing metabolic substrate as precursors for biosynthetic reactions. While the demand for mitochondrial ATP production is decreased in tumour cells, the requirement for biosynthetic precursors and reducing power in the form of nicotinamide adenine dinucleotide phosphate is enhanced. In order to maintain mitochondrial function under limited pyruvate availability due to the increased rate of glycolysis, tumour cells often rely on increased glutaminolysis.^72^ Glutamine, the most abundant circulating amino acid,^73^ is transported into the cell and converted to glutamate and further to α-KG to replenish the TCA cycle. Indeed, strategies to inhibit glutaminolysis have proven effective in slowing the proliferation of cancer cells.^74^ Addiction to glutamine is a prominent feature of Kirsten rat sarcoma virus, oncogene (KRAS)-mutant cancers.^75,76^ A recent study showed that mutant KRAS rewires glutamine metabolism to support succinate biosynthesis from α-KG in colorectal cancer cells, resulting in an overall reduction of 5 hmc and CpG hypermethylation, and the activation of Wingless-related integration site/β-catenin signalling.^77^

### 2.4 Cellular metabolism influences histone acetylation

More than 30 years passed between the first description of histone tail acetylation^78^ and the discovery of histone acetyltransferases (HATs) and histone deacetylases (HDACs) that add and remove this modification respectively.^79,80^ Histone acetylation almost exclusively marks chromatin for transcriptional competency by two main mechanisms: (i) by changing the overall charge on the histone tail, which disrupts the binding of nucleosomal core components and renders the DNA accessible to transcriptional machinery and (ii) by acting as recruitment sites for various nucleosome remodelling proteins and transcription initiation factors that contain bromodomains—motifs that bind acetylated lysines.^81^

HATs transfer an acetyl group from acetyl-CoA to lysine residues on histone tails. Acetyl-CoA is produced by the oxidative decarboxylation of pyruvate from glycolysis by the pyruvate dehydrogenase complex (PDHC), the oxidation of long chain fatty acids, or the oxidative degradation of certain amino acids. Therefore, its abundance is dependent on glucose availability, fatty acid oxidation, and mitochondrial respiratory function.^82^ While histone acetylation is highly regulated, often in a gene-selective manner the Michaelis constant (Km) of most HATs falls within the range of cellular acetyl-CoA concentrations.^83^ As a consequence, the availability of acetyl-CoA can restrict or promote global levels of histone acetylation. Therefore, changes to metabolic pathways that influence acetyl-CoA levels potentiate large-scale changes in gene expression. And because glucose flux dose-dependently regulates histone acetylation,^84^ the acceleration of glycolysis in cancer is associated with global histone hyperacetylation.^85^ Accordingly, a glucose derivative that inhibits hexokinase and thereby blocks the first step of glycolysis, 2-deoxyglucose, significantly lowered acetyl-CoA levels and suppressed the acetylation of all four core histones in multiple cancer cell lines. For a broader discussion of the various metabolic sources of acetyl-CoA that support histone acetylation, we recommend a recent article by Feron.^86^

Interestingly, functional PDHC is also detected in the nucleus where it locally supplies acetyl-CoA to histone acetylation reactions.^87^ Moreover, nuclear PDHC levels were shown to increase in a cell cycle-dependent manner and in response to serum, growth factors or mitochondrial stress. This was concomitant with a decreased mitochondrial PDHC levels, suggesting that PDHC translocates from mitochondria to the nucleus to link acetyl-CoA synthesis with epigenetic regulation.^87,88^

### 2.5 NAD^+^-dependent chromatin modifiers

The mammalian HDACs discovered so far are divided into four classes based on sequence homology and domain organization.^89^ The class III HDACs, which are homologous to the yeast silent information regulator 2 (Sir2) and known as sirtuins in mammals, are most closely connected with metabolism. Sirtuins are a family of nicotinamide adenine dinucleotide NAD^+^-dependent deacetylases associated with longevity.^90^ In addition, there is evidence that some members of the sirtuin family can also catalyse the mono-adenosine diphosphate (ADP)-ribosylation of histones.^91^ While sirtuins play important roles in numerous biological processes including cellular metabolism,^92^ understanding their complex functions and dual characteristics as both promoters and suppressors of malignant characteristics in different cancers remains a significant challenge.^93^

The ADP-ribosyltransferases (ARTs), formerly known as poly ADP-ribose polymerases (PARPs), catalyse the transfer of one or more ADP-ribose groups from NAD^+^ to target proteins (at arginine, asparagine, cysteine, and histidine amino acids), including histones. ADP-ribosylation of histones and other nuclear proteins is predominantly associated with nucleosome remodelling in DNA repair.^94–96^ In response to extreme DNA damage, ADP-ribosylation can deplete NAD^+^ levels in the cell to the point where ATP production and other aspects of cellular metabolism are interrupted. Despite several recent studies indicating that ADP-ribosylated histones also have important roles in proliferation, replication, and transcription (recently reviewed^97^), knowledge of the chromatin-dependent function of this modification remains limited. Mono-ADP-ribosylation of H3 histones at arginine 117 (H3R117) was recently characterized as a modification associated with the proliferation of colorectal cancer cells.^98^ Li *et al*.^99^ demonstrated that mono-ADP-ribosylation of H3R117 limited local poly-ADP-ribosylation of *TET1* promoter in human colon adenocarcinoma cells. This was associated with enrichment of 5 mc and depletion of H3K4me3 at the *TET1* promoter, which culminated in a reduction of *TET1* transcription. Moreover, this down-regulation of *TET1* expression impaired TET1-dependent demethylation reactions to epigenetically silence the *TFPI2* tumour suppressor gene.

## 3. Immuno-metaboloepigenetics

Over the past decade, immunologists have increasingly developed an appreciation for metabolism.^100^ Highly sensitive techniques to measure flux through metabolic pathways, metabolomic approaches that show how metabolites are directly connected to immune cell function, and the application of new pharmacological tools to models of infection and inflammation have driven the emergence of the field of immunometabolism.^24,100^ Central to this burgeoning area of research is the observation that immune cells with different functions engage distinct metabolic pathways.

As described in the previous section, glycolysis is less efficient than OxPhos for generating ATP from glucose. However, immune cells that require rapid production of ATP will switch to glycolysis.^24^ Glycolysis is enhanced in adaptive immune cells (slow-acting, long-term defence) such as activated effector T cells^101^ and activated B cells,^102^ as well as innate immune cells (rapid, front-line defence) such as activated dendritic cells^103^ and activated natural killer cells.^104^ Macrophages are also particularly interesting innate immune cells in this respect, as glycolytic metabolism can distinguish macrophage polarization subsets induced by anti-inflammatory [interleukin (IL)-4]^105^ or pro-inflammatory [bacterial endotoxin lipopolysaccharide (LPS)] stimuli. Inflammation resolving macrophages (M2 polarization state) are characterized by enhanced fatty acid oxidation^106^ and a reliance on OxPhos.^107^ In contrast, enhanced glucose uptake and glycolysis are metabolic traits of pro-inflammatory (M1 polarization state) macrophages.^108^ Another key difference concerns the TCA cycle, which is coupled with OxPhos in M2 macrophages, but is broken in two places in M1 macrophages. This leads to the accumulation of citrate and succinate and a down-regulation of OxPhos under aerobic conditions.^108,109^ The switch to Warburg metabolism is directly associated with pro-inflammatory cytokine production through the inhibition of prolyl hydroxylases by succinate, which stabilizes HIF1α and sustains IL-1β production.^109^

Driven by this strong connection between metabolism and immune function, immunologists have begun to delineate the functional epigenetic effects of these metabolic changes in macrophages. M2 macrophages accumulate α-KG through increased glutaminolysis.^108,110^ Mechanistically, the accumulation of α-KG is important for JMJD3-dependent demethylation of H3K27me3 in M2 gene induction.^110^ Furthermore, this study identified the importance of the α-KG/succinate ratio for the induction of distinct macrophage activation states: a high α-KG/succinate ratio promotes M2 activation, whereas a low α-KG/succinate ratio supports the pro-inflammatory phenotype of M1 macrophages. Although it is a relatively new area of research for which there is limited experimental evidence thus far, understanding how immune responses are orchestrated through metaboloepigenetic reprogramming offers novel insight into mechanisms supporting the broad spectrum of macrophage phenotypes. In this section, we discuss the current knowledge of metaboloepigenetic mechanisms associated with innate immune memory in macrophages.

### 3.1 Trained (innate) immunity

**Figure 2:**
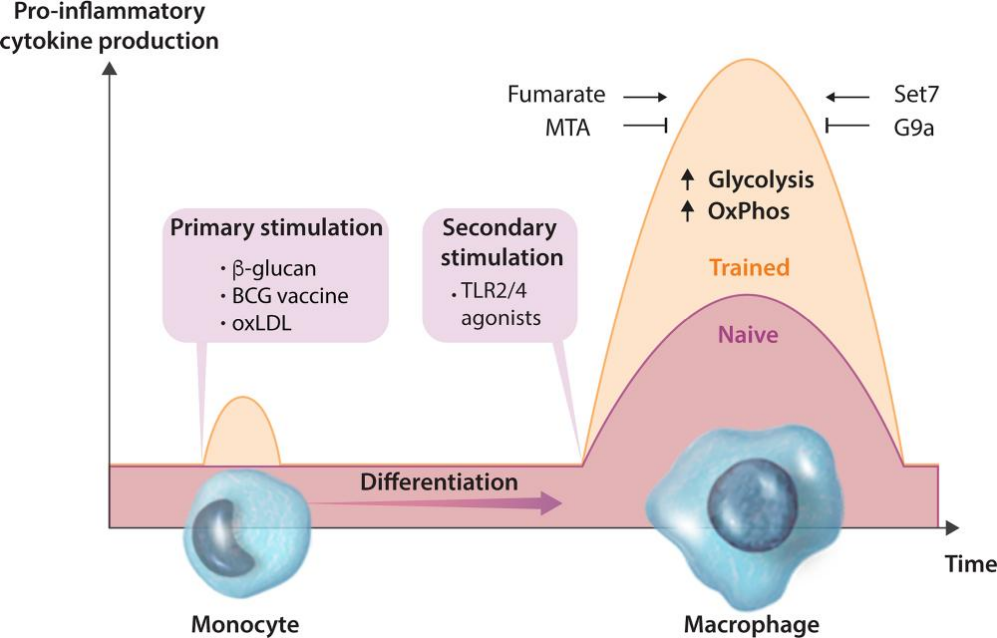
Trained immunity is dependent on metabolic and epigenetic changes. Innate immune cells, such as monocytes, can be functionally reprogrammed in response to exogenous or endogenous stimuli, leading to an altered immune response to a second, unrelated challenge after the return to a non-activated state. Primary stimulation with β-glucan, BCG vaccine or oxLDL induces a de facto memory that is revealed by secondary stimulation with agonists of TLR 2 and 4. Trained immunity is characterized by upregulation of glycolysis and OxPhos, as well as the accumulation of fumarate. The augmented cytokine production is abolished by the global methyltransferase inhibitor 5′-deoxy-5′-MTA and specific inhibition of Set7. Inhibition of G9a enhances the trained phenotype.

Contrasting the traditional dogma that memory is the proprietary of the adaptive immune system, the sensitivity of innate immune cells to Toll-like receptor (TLR) stimulation can be programmed by a phenomenon called ‘trained immunity.’^111^ Trained immunity is best characterized in monocyte-derived macrophages, although it has also been described in dendritic cells^112^ and natural killer cells.^113^ When primary monocytes are stimulated with certain microorganisms or microbial ligands, they differentiate into *trained* macrophages that have the capacity to respond to TLR agonists or other pathogen-associated molecular patterns with heightened pro-inflammatory cytokine production (*Figure 2*). Importantly, the secondary stimuli can be entirely unrelated to the first. Therefore, the effects of trained immunity are considered to be non-specific.^114^

Trained immunity is exemplified by the augmented production of tumour necrosis factor (TNF) α and IL-6 induced by *Candida albicans* or its cell wall component β-glucan in human macrophages^115,116^ and mice that lack mature B and T cells.^117^ More recently, trained immunity induced by β-glucan was shown to be protective against infections by *Leishmania braziliensis*^118^ and *Mycobacterium tuberculosis*.^119^ Similarly, the induction of trained immunity by the Bacille Calmette–Guérin vaccine (BCG) is the most likely explanation for its heterologous protection against experimental yellow fever.^120^ In the clinic, trained immunity is the proposed mechanism through which BCG exerts its protective effects against a wide range of infections in newborns,^121^ as well as its anti-tumour properties in the treatment of bladder cancer.^122^ On the other hand, monocytes exposed to a low concentration of LPS differentiate into *tolerant* macrophages that are refractory to TLR restimulation. Also a type of trained immunity, endotoxin tolerance is a major cause of immunosuppression induced by Gram-negative sepsis.^123^

#### 3.1.1 Epigenetic rewiring in trained immunity

Much of what we know about trained immunity has been learned from studying a standard cellular model that involves the training of human peripheral monocytes, in which these cells are exposed to a stimulus for a short period of time (usually 24 h). The cells are then incubated for 5–7 days during which they differentiate into macrophages. At this point, trained macrophages exhibit a heightened response to heterologous secondary stimuli (*Figure 2*).^116^ β-glucan-trained, naïve, and tolerant macrophages are distinguished by strikingly divergent genome-wide H3K4me1, H3K4me3 and H3K27ac.^124,125^ Many of these epigenetic changes are established early in the programme of training or tolerance and persistent H3K4me1 and H3K4me3 marks are considered central to the transcriptional memory.^124,125^

Co-incubation with the non-specific methyltransferase inhibitor 5′-deoxy-5′-methylthioadenosine (MTA) during the first 24 h of *in vitro* training completely nullifies the augmented cytokine response mediated by BCG^117^ and β-glucan.^116,119,126^ A proportion of these methyl events are regulated by the H3K4me1 writer Set7, which exhibits increased expression and activity in response to β-glucan.^116^ Co-administration of specific Set7 inhibitors prevented the induction of trained immunity by β-glucan and BCG in human macrophages. Furthermore, Set7 knockout mice were unable to mount β-glucan-mediated trained immunity against endotoxin challenge.^116^ In contrast, expression of G9a, a KMT that writes the repressive H3K9me2 modification, is decreased in cells trained with β-glucan. Specific inhibition of G9a reduced H3K9me2 at promoters of genes involved in trained immunity and simultaneously amplified trained immunity responses.^127^

### 3.2 The immunometabolic-epigenetic axis of trained immunity

Altered metabolism could partly account for wide-spread changes in histone acetylation in trained immunity.^124,125^ A key metabolic hallmark of trained cells is enhanced glycolysis via activation of the Akt-mTOR-HIF1α pathway.^115^ The trained phenotype is dependent on this glycolytic metabolism insofar as the augmented cytokine response is nullified by pharmacological inhibition of key enzymes involved in glycolysis.^18,115,128^ While concentrations of acetyl-CoA were unaltered in trained immunity,^18^ changes to the NAD^+^/NADH ratio^115^ indicated that sirtuins could be involved in establishing histone acetylation patterns in trained cells. So far, only one study has investigated this link, recently demonstrating that SIRT1 has a minor and perhaps redundant role in trained immunity.^129^ Another source of NAD^+^ is the breakdown of pyruvate to lactate that occurs in aerobic glycolysis and is consequently observed in trained immunity.^116^ Intersection of metabolome and transcriptome data and subsequent pharmacological studies revealed that the cholesterol synthesis pathway is also indispensable for trained immunity induced by β-glucan and BCG.^19,130^

Initial characterization of β-glucan-induced trained immunity revealed a metabolic shift towards glycolysis at the expense of OxPhos.^27^ However, recent studies show that macrophages trained with a lower concentration of β-glucan (1 ug/mL instead of 10 ug/mL) maintain a functioning TCA cycle that generates increased amounts of ATP by OxPhos while also providing metabolites that potentially modulate inflammatory functions.^116^ Genetic variation in TCA cycle genes such as *IDH* and *SDH*, as well as the NADH dehydrogenase subunit of complex I of the electron transport chain *NDUFB7*, is associated with variation in β-glucan-trained cytokine production. The reasons why different β-glucan concentrations induce opposite effects on OxPhos remain unclear, however the up-regulation of both glycolysis and OxPhos is consistent with the metabolic phenotypes of macrophages trained with BCG^18^ or endogenous training stimuli^128,131,132^ (discussed later in the Review).

Chief among the altered TCA cycle metabolites in trained immunity is fumarate, which accumulates in cells trained with β-glucan and integrates epigenetic, immune and metabolic circuits by inhibiting the KDM5 family of H3K4 demethylases.^19^ This could partly account for the enrichment of H3K4me3 observed in trained immunity throughout the genome and particularly at the promoters of *TNF* and *IL6*. Perhaps most convincing however is the observation that fumarate itself can induce trained immunity and partially recapitulate the enrichment of H3K4me3 induced by β-glucan at the *TNF* and *IL6* promoters and other key regulatory sites. In addition to fumarate, the concentrations of 2-HG and succinate, which similarly antagonize α-KG-dependent KDMs, was also increased in β-glucan-trained macrophages.^19,116^ This was paralleled by increased expression of *SDH* genes, which can account for the enhanced conversion of succinate to fumarate. Fumarate-dependent inhibition of KDM5 activity was restored by the addition of α-KG, which also partially counteracted the training effect of fumarate on cytokine production.^19^

### 3.3 The chromatin is SET for metabolic rewiring in trained immunity

The accumulation of fumarate and malate, as well as the up-regulation of *SDHB* in β-glucan-trained macrophages is abolished by pharmacological inhibition of Set7 during the first 24 h of training *in vitro*. Mechanistically, this effect is partly explained by Set7-dependent H3K4me1 enrichment at distal enhancers that topologically associate with the *SDHB* promoter.^116^ Considering the metaboloepigenetic effects of fumarate in trained immunity,^19^ this study identifies a potential mechanism by which Set7 influences H3K4 methylation not only through its own methyltransferase activity, but also by indirectly inhibiting histone demethylation. SDH is unique in its participation in both the TCA cycle and the mitochondrial electron transport chain. Accordingly, the increase in OxPhos induced by β-glucan training was abolished by Set7 inhibition.^116^ This crucial role of Set7 early in the induction of trained immunity is consistent with its role as an epigenetic writer of metabolic memory in cultured vascular endothelial cells^133,134^ and peripheral blood mononuclear cells from patients with type 2 diabetes mellitus (T2DM).^135^

## 4. Metaboloepigenetics in CVD

Infectious diseases have been replaced by CVD as the leading cause of death globally over the last 20 years, with atherosclerosis the main underlying cause.^105^ The development and progression of atherosclerosis, which involves the metabolically driven activation and remodelling of vascular and immune cells,^136^ is increasingly explored in an epigenetic context.^137,138^ The acceleration and increased incidence of atherosclerosis in diabetes points further towards the involvement of metaboloepigenetic processes in atherosclerotic CVD.

### 4.1 Maladaptive trained immunity in atherosclerosis

While clearly beneficial for fighting infections, trained immunity is a double-edged sword when it comes to inflammatory diseases where innate immune cells are the proponents of tissue injury. Monocytes isolated from patients with an increased risk for atherosclerotic CVD due to elevated LDL-cholesterol levels^139^ and patients with severe coronary atherosclerosis^140^ exhibit augmented cytokine production capacity *ex vivo*. Furthermore, several endogenous compounds that accelerate atherosclerosis, including oxidized LDL (oxLDL),^128,141^ lipoprotein(a),^142^ aldosterone,^143^ epinephrine, and norepinephrine^131^ induce trained immunity in human macrophages. Apart from aldosterone, each of these stimuli induce a metabolic phenotype like BCG and β-glucan training: concurrent up-regulation of glycolysis and OxPhos. In the case of oxLDL, pharmacological inhibition of glycolysis prevented the induction of trained immunity and prevented H3K4me3 enrichment at the *TNF* and *IL6* promoters.^128^ Precisely, how glycolysis influences histone lysine methylation events in trained immunity remains untested. One potential mechanism is that the glycolytic production of acetyl-CoA stimulates H3K14 acetylation,^84^ which can inhibit LSD1-mediated demethylation of H3K4me3.^144^

Atherosclerosis is characterized by chronic, low-grade sterile inflammation. This implies a long-term activation of the innate immune system despite the short lives of circulating innate immune cells. Feeding a high cholesterol Western diet (WD) for 4 weeks to atherosclerosis-prone *Ldlr*^−/−^ mice induced trained immunity that persisted when the mice were switched back to a chow diet for 4 weeks.^145^ This metabolic memory occurred in the bone marrow niche and was dependent on the skewing of haematopoietic stem cells (HSCs) towards myelopoiesis. Indeed, hypercholesterolemia is linked to the reprogramming of HSCs^146^ and an increase in circulating monocytes correlates with cardiovascular events.^147^ Moreover, inflammatory monocytes that derive from activated haematopoietic precursors during WD feeding differentiate into atherogenic macrophages.^148^ The induction of trained immunity in the WD-fed *Ldlr*^−/−^ mice was dependent on activation of the NLRP3 inflammasome. This is important because similar induction of trained immunity and myelopoiesis in the bone marrow of wild-type mice administered β-glucan was associated with increased IL-1β production, a key inflammasome-mediated product.^149^ Together, these findings underscore IL-1β as a central endogenous mediator of trained immunity *in vivo*. Indeed, the Canakinumab Antiinflammatory Thrombosis Outcome Study trial demonstrated the clinical benefit of IL-1β blockade for cardiovascular risk.^150^ Interestingly, increased expression of *Il1b* in the bone marrow of mice trained with β-glucan was significantly reduced in animals lacking Set7, suggesting a role for this KMT in the long-term activation of trained immunity.^116^ Further exploration of metaboloepigenetic changes in endogenous trained immunity could yield novel approaches to interfere with the development and progression of atherosclerosis.

### 4.2 Metaboloepigenetics in vascular complications of diabetes

Vascular complications are the major cause of the clinical, social and economic burden of type 1 diabetes mellitus (T1DM) and T2DM.^151,152^ Diabetes accelerates atherosclerosis and more than doubles the risk of CVD, which further increases with worsening glycemic control.^153^ Hyperglycemia can alter the epigenetic landscape of the microvasculature and the macrovasculature in diabetes, which may precede cardiovascular complications.^154^ The metabolic perturbations of the diabetic milieu are fertile ground for the discovery of metaboloepigenetic connections to atherosclerosis.

Hyperglycemia is associated with changes in histone acetylation in vascular cells. Genome-wide analysis of monocytes isolated from T1DM patients revealed elevated levels of H3K9ac enriched at gene promoters related to NFκB signalling and diabetes complications.^155^ Importantly, H3K9ac was significantly associated with mean levels of glycated haemoglobin—a marker of recent blood glucose levels. High glucose (HG) induced genome-wide histone hyperacetylation in human aortic endothelial cells and the specific induction of genes and pathways associated with endothelial dysfunction through the enrichment of H3K9/K14ac.^156^ Genome-wide histone hyperacetylation requires a substantial amount of acetyl-CoA as substrate for HATs in the nucleus. In microvascular cells of the kidney, HG induces the expression and activity of ATP-citrate lyase (ACL),^157^ which catalyses the synthesis of acetyl-CoA and oxaloacetate from citrate.^158^ Moreover, HG promotes the nuclear translocation of ACL to support the increased demand for acetyl-CoA for histone hyperacetylation.^159^ The increased demand for acetyl-CoA requires an increased supply of citrate, which comes from enhanced glycolysis driven by HG.^157^

Hyperglycemic memory is a phenomenon that has received considerable research attention. Prior exposure to HG can induce gene expression changes that persist even after the restoration of normal glucose conditions.^15^ Hyperglycemic memory occurs in human vascular endothelial cells^133,134^ as well as mouse bone marrow-derived macrophages (BMDMs), where it promotes M1-type responses and suppresses M2-type responses.^160^ Bone marrow obtained from diabetic mice and transplanted into normoglycemic atherosclerosis-prone mice retained a memory of its previous hyperglycemic environment to drive accelerated atherosclerosis and increase plaque macrophage content.^161^ BMDMs from diabetic mice exhibited a trained immunity phenotype, with enhanced *Il6* and M1-associated gene expression following stimulation with LPS and IFN-γ. Hyperglycemia-induced trained immunity in HSCs was driven by epigenetic reprogramming of H3K4me3 and H3K27ac, which was likely to be downstream of metabolic changes, however a causal link was not established. HG exacerbates the training effect of oxLDL in terms of pro-inflammatory cytokine production capacity,^128^ which could be associated with the acceleration of atherosclerosis in diabetes.^162^ Indeed, exposure of peripheral monocytes to HG enhances the polarization of macrophages towards an M1-like phenotype, which correlates with increased succinate production and its potential to interfere with epigenetic reactions.^161^ To this end, Green and Brewer^163^ recently suggested that dysregulation of α-KG-dependent dioxygenases by hyperglycemia could link diabetes with vascular disease.

## 5. Future directions: metabolites impact the posttranslational regulation of TFs and epigenetic enzymes

So far, we have focused on the role that metabolites play in the modification of DNA and histones. However, many non-histone proteins are also post-translationally modified by the same machinery. In this section, we briefly describe some important considerations for interpreting the effects of metabolism on gene expression. The significance to the overall discussion is that metabolic changes could have a major impact on gene expression by modulating the transcriptional machinery in addition to chromatinized proteins.

Whenever there is a discussion of epigenetics, the elephant in the room is invariably the TF. Their power to activate or repress transcription is demonstrated by numerous cell-reprogramming experiments,^164^ best exemplified by a set of experiments showing that the forced expression of four specific TFs can reprogramme fibroblasts to stem cells.^165^ Several enzymes that mediate PTMs have dual roles modifying histones and non-histone proteins, and every modification that can occur on histones can also be written to non-histone proteins. Thousands of these modification sites have been identified across the proteome, and many have been shown to modulate protein function and stability.

Owing to advances in affinity enrichment methods and mass-spectrometry-based proteomics,^166,167^ methylation has emerged as a critical regulator of non-histone protein function.^168^ Numerous KMTs and KDMs target TFs with comparable affinity to their histone substrates, making the transcriptional events regulated by these enzymes difficult to interpret. Set7 has the most non-histone substrates identified so far,^169^ with the search for new substrates based on the presence of motifs commonly flanking targeted lysines.^170,171^ HIF1α is methylated at K32 by Set7, resulting in the modulation of HIF1α occupancy of target gene promoters in fibroblasts.^172^ In response to IL-6, STAT3-dependent *SOCS3* expression in colon cancer cells is regulated by Set7-mediated methylation of STAT3 at K140, and the methyl groups are removed by LSD1.^173^ The gene-regulating activity of the p65 subunit of NFκB, which has roles in cancer, immune regulation and CVD,^174^ is modulated by lysine methylation mediated by Set7^175,176^ and the H3K36 methyltransferase NSD1, with the latter being antagonized by KDM2A.^177^ From these examples, it is not difficult to imagine how large-scale metabolic changes, such as changes to SAM/SAH ratios, FAD^+^ availability, or α-KG/succinate ratios could influence the activity of TFs. Similar metabolome-PTM crosstalk potentially influences TF acetylation, which has also been shown to regulate TF activity and stability,^178^ through changes in acetyl-CoA and NAD^+^/NADH ratios.

Also important to transcription is the intricate system of functional PTMs written (and erased) by epigenetic enzymes to other epigenetic enzymes. Again, the prototypical example is Set7. The transcriptionally-repressive HMT activity of SUV39H1, a H3K9 methyltransferase associated with heterochromatin formation, is down-regulated when SUV39H1 is methylated at K105 and K123 by Set7.^179^ Set7 can also support transcriptional activation by directing the lysine methyl-dependent degradation of DNMT1^180^ and by methylating the PCAF HAT at multiple sites.^181^ Furthermore, Set7-dependent methylation of ARTD1 (formerly PARP1) stimulates the synthesis of poly-ADP-ribose in response to oxidative stress.^182^ And like Set7, ARTD1 itself can maintain transcriptionally permissive chromatin structure by (i) modulating DNMT1 activity through ADP-ribose polymers and by (ii) preventing KDM5B-dependent demethylation of H3K4me3.^183^ Similar epigenetic enzyme modulating events are also associated with lysine acetylation. For example, autoacetylation by the p300 is important for stimulating its HAT activity.^184^ We previously described how pharmacological HDAC inhibition shows a complex pattern of gene expression changes that were associated with histone acetylation and histone deacetylation events, suggesting that HDAC inhibition could interfere with PTM of HATs and/or other HDACs.^15,185^

## 6. Concluding remarks

This Review is designed to stimulate interest in this emerging field of metaboloepigenetic mechanisms of gene regulation. The growing literature on the influence of intracellular metabolites on epigenetic reactions in cancer cells continues to provide new insights into mechanisms of gene regulation relevant to other diseases and biological processes. The metabolic profiles of M1-like macrophages and trained macrophages bear some resemblance to cancer cells, particularly with regard to the up-regulation of glycolysis and glutaminolysis. The metaboloepigenetic effects of these and other pathways can potentially be exploited for the improvement of vaccine strategies. On the other hand, a similar strategy could be employed to attenuate atherogenic innate immune cells. A deeper knowledge of the ways that metabolic changes reverberate through TF and chromatin-modifying networks to reprogramme gene expression is anticipated to significantly advance our understanding of metaboloepigenetics in health and disease.

## Data Availability

The data underlying data are incorporated into the review article.
